# A functional variant in the *UBE2B* gene promoter is associated with idiopathic azoospermia

**DOI:** 10.1186/s12958-015-0074-4

**Published:** 2015-07-30

**Authors:** Lisha Mou, Qiang Zhang, Ruiying Diao, Zhiming Cai, Yaoting Gui

**Affiliations:** Shenzhen Domesticated Organ Medical Engineering Research and Development Center, First Affiliated Hospital of Shenzhen University, Shenzhen, China; Guangdong and Shenzhen Key Laboratory of Male Reproductive Medicine and Genetics, Institute of Urology, Peking University Shenzhen Hospital, Biomedical Research Institute, Shenzhen PKU-HKUST Medical Center, Shenzhen, China; The people’s hospital of Ankang, Shanxin, China

**Keywords:** Male infertility, Idiopathic azoospermia, UBE2B, SP1

## Abstract

**Background:**

A variety of genetic variants lead to abnormal human spermatogenesis. The ubiquitin-conjugating enzyme E2B (*UBE2B*) plays a significant role in spermatogenesis as *Ube2b*-knockout male mice are infertile.

**Methods:**

In this study, we sequenced the exon and promoter region of *UBE2B* in 776 patients diagnosed with idiopathic azoospermia (IA) and 709 proven fertile men to examine whether *UBE2B* is involved in the pathogenesis of IA.

**Results:**

In the exon region, two novel synonymous variants were detected in the patient group. In the promoter region, four known variants and four novel variants were identified in the patient group. Of the novel variants in the promoter region, three were located at the binding site of specificity protein 1 (SP1) transcription factor analyzed by TRANSFAC software. Luciferase assays demonstrated that one heterozygous variant (Chr5.133706925 A > G) inhibited the transcriptional regulation activity of SP1.

**Conclusions:**

A novel variant (Chr5.133706925 A > G) residing in the *UBE2B* gene promoter region confers a high risk for IA in a Chinese population. These results support a role for *UBE2B* in the pathogenesis of IA.

## Background

Approximately 15 % of couples at child-bearing age are infertile [1–3]. Idiopathic azoospermia (IA), one of the most severe forms of male infertility, affects up to 1 % of all adult men in the general population [4]. Although the genetic causes of IA remain largely unknown in humans, familial case reports and experimental studies in mice have demonstrated that many variants in different genes can result in spermatogenesis defects [5–7]. Recent studies also indicate that genetic variants in the promoter region are associated with IA risk as well [8–10].

The ubiquitin-conjugating enzyme E2B (UBE2B), also known as Rad6b or Hr6b, is located at chr5:133706870–133727799. UBE2B belongs to the Ubiquitin Proteasome System, which mediates H2A and H2B ubiquitylation and function during transcription [11]. Previous studies have shown that the variants of UBE2B are associated with male infertility in humans [12–15]. Other evidence from experimental studies has also shown that *Ube2b* knockout male mice are infertile with low numbers of predominantly abnormal spermatozoa, abnormal spermatid nuclear condensation, sperm periaxonemal anomalies, damaged synaptonemal complex structure and/or increased cross-over frequency [16, 17].

Because the *UBE2B* gene is essential for normal spermatogenesis [12–14, 18], the genetic variants that affect UBE2B expression may be involved in the etiology of human IA. Therefore, the objective of this study was to determine the association between *UBE2B* gene variants and male infertility.

## Methods

### Ethical approval

The study was approved by the ethics committee of Peking University Shenzhen Hospital. The approval reference number is 20090018. The study was approved on July 18th, 2009, initiated on August 1st, 2009 and terminated on December 1st, 2014.

### Patient samples

A total of 1,880 azoospermic patients were recruited for this study from the Peking University Shenzhen Hospital and the Center of Reproductive Medicine, Tongji Medical College, Huazhong University of Science and Technology. Among them, 776 patients fulfilled the criteria for IA diagnosis as follows: (1) no sperm detected in the pellets of semen samples at three different occasions, (2) no obstruction, inflammation and injury of the reproductive system or pelvic cavity, (3) no endocrinological defect, and (4) no karyotypic abnormality and Y chromosome microdeletion. A total of 709 fertile men from the Center of Physical Examination, Peking University Shenzhen Hospital were recruited as controls: they had fathered at least one child without assisted reproductive techniques such as IVF, ICSI and IMSI. After a panel re-sequencing study and quality control steps, 776 patients aged 24–46 years (average 30.6) and 709 fertile men aged 29–51 years (average 35.6) were available for further analysis. Informed written consent was obtained from each subject.

### Panel re-sequencing study

Five micrograms of genomic DNA isolated from peripheral blood samples were sent to the Beijing Genomics Institute at Shenzhen for exome capture and sequencing. The capture procedure was performed in solution with a NimbleGen custom array (Roche NimbleGen, Madison, WI, USA) that is capable of enriching the exonic sequences of 654 infertility- or subfertility-related genes [19]. Most of these genes were reviewed by Matzuk and Lamb [5]. Moreover, we also selected other genes that were shown to cause male reproductive defects in mouse models from studies published between November, 2008 and December, 2010. A panel re-sequencing study was performed on the Illumina platform with pair-end 90 bp reads.

Fastq sequence files were aligned against the human reference genome (NCBI build 37.1, hg19) with the SOAPaligner software (2.21). Duplicated pair-end reads were removed from the merged data sets. Single nucleotide variants that were different from the hg19 reference genome were filtered out if they met any of the following criteria: Phred-like quality score ≤ 20, overall depth ≤ 8×, estimated copy number ≥ 2 or the genomic distance between two adjacent variants < 5 bp. In addition, the quality score of both the major and minor allele at heterozygous locus should be at least 20. Variants were then annotated using an in-house functional prediction tool and were compared to dbSNP135 and 1000 Genomes databases (as of August, 2010).

### Polymerase chain reaction (PCR) analysis of UBE2B promoter

Four sets of PCR primers (Table 1) were developed and optimized to amplify the promoter and 5’ UTR region 2551 bp upstream of the *UBE2B* coding region from HeLa cells by PCR with the following primers: promoter 1, CCCAGGACAAAGATGAAC (forward), AGAATCGCTTGAGACGAG (reverse); promoter 2, CTTCAACTTCCTCGTCTCA (forward), CACCGTCCTTCCCTCTAT (reverse); promoter 3, AGAGGGAAGGACGGTGAC (forward), CGGGTTTAAGAGGGTGAG (reverse); and promoter 4, CCTCTTGGGTAATGTTGTC (forward), CATCTAGGCGAAGGTGAA (reverse).

Forward and reverse amplification primers were also used as sequencing primers. Sequencing was conducted on an ABI 3730 DNA analyzer. Variants were then annotated using an in-house functional prediction tool and were compared to dbSNP135 and 1000 Genomes databases (as of August, 2010).

### Plasmids construction

The wild type (WT) and mutant *UBE2B* promoter bearing one of the identified variants (Chr5.133706771 T > A, Chr5.133706876 T > G, Chr5.133706925 A > G) were amplified by PCR with the primers 5'-CGGGGTACCAGGGGCAAGGCTGAGGCAATGTT -3' (forward) and 5'-CTAGCTAGCAGGGGCGGGCGGATAATGTCTGAT -3' (reverse) from the patient genome. The PCR products were subcloned into a psiCHECK™-2 vector (C8021, Promega, Madison, WI) with KpnI/NheI sites. All clones were verified by DNA sequencing.

### Luciferase assay

HeLa, 293FT and TM4 cells (ATCC, Manassas, VA, USA) were cultured in Dulbecco’s Modified Eagle’s Medium (Gibco BRL, Gaithersburg, MD, USA) supplemented with 10 % fetal bovine serum, 100 U/mL penicillin and 100 μg/mL streptomycin at 37 °C, 95 % humidity and 5 % CO_2_. Cells were seeded in 24-well tissue culture plates 24 h prior to transfection. Transfection was performed using Lipofectamine 2000 (Invitrogen, Carlsbad, CA, USA) according to the manufacturer’s instructions. Equivalent amounts (500 ng) of pCMV- SP1 (OriGene, Rockville, MD, USA), pCMV empty vector were cotransfected with wild type (WT) or mutant *UBE2B* promoter (Chr5.133706771 T > A, Chr5.133706876 T > G, Chr5.133706925 A > G) plasmids (250 ng), respectively. Cells were harvested 36 h after transfection and assayed for firefly and *Renilla* luciferase expression using the Dual Luciferase Reporter Assay System (Promega). *Renilla* luciferase activities were normalized to firefly luciferase activity.

### Histopathology

Testicular tissues from patients with azoospermia were collected and immediately stored at 4 % paraformaldehyde solution. The tissues were sent to the pathology department for sections. Slides were stained with hematoxylin and eosin and then examined using light microscopy.

### Statistical analysis

Data were expressed as the mean ± SD. The Student’s *t*-test with Bonferroni correction was used to compare the difference in mean between the variants and WT. *P* < 0.05 was considered statistically significant.

## Results

### Identification of UBE2B variants in patients with IA

To examine whether *UBE2B* genetic defects were associated with IA, we screened for *UBE2B* exonic variants in 776 IA patients and 709 men with proven fertility using the massively parallel sequencing technology. As shown in Table 1, two synonymous variants were detected in the *UBE2B* exon region. These two variants were not reported as genetic polymorphisms in the public database dbSNP135 or in the 1000 Genome Project dataset. However, no missense variants were identified in our study.

**Table 1:** *UBE2B* exonic variants in IA patients and controls

No.	Chr5.position	Sequence variants	Patients (n = 776)	Control (n = 709)	Function
1	133712363	A > G	1	0	Synonymous variant
2	133724047	C > A	0	2	Synonymous variant

**Fig. 1:**
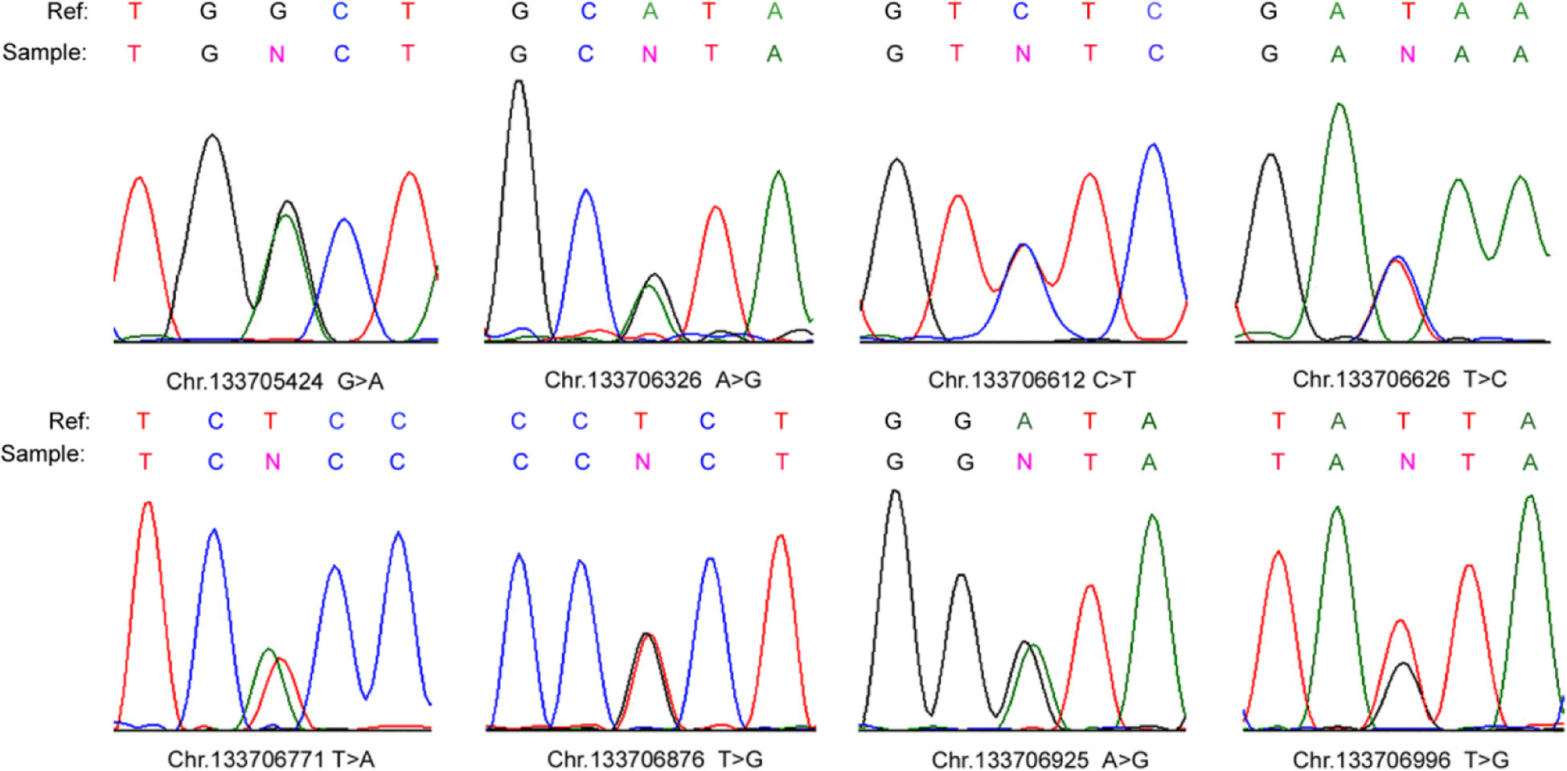
Eight variants of the *UBE2B* promoter in patients with IA. Chromatogram traces from Sanger sequencing showing the variants. Ref: reference sequences

To examine whether *UBE2B* promoter variants were associated with IA, we further screened for *UBE2B* promoter variants by PCR and Sanger sequencing (Fig. 1). Sequence analysis of the *UBE2B* promoter region in 776 IA patients and 709 men with proven fertility revealed 8 variants (Table 2). As shown in Table 2, four variants had not been reported in either the dbSNP135 database or the 1000 Genome Project dataset and were found to be absent in the normal controls. Further analysis by TRANSFAC software showed that three of these variants (Chr5.133706771 T > A, Chr5.133706876 T > G, and Chr5.133706925 A > G) were located at the binding site of the SP1 transcription factor. We hypothesized that these three variants may affect the DNA-binding ability of SP1 to the *UBE2B* promoter, thus modifying UBE2B expression. The following study was focused on these three variants. In particular, one of these three variant (Chr5. 133706925 A > G) was identified in 32 patients. All of the 32 patients were heterozygous variants.

**Table 2:** *UBE2B* promoter variants in IA patients and controls

No.	Chr5.position	Sequence variants	Patients (n = 776)	Control (n = 709)	dbSNP135
1	133705424	G > A	4	0	rs191553312
2	133706326	A > G	3	6	rs36087117
3	133706612	C > T	12	15	rs1379247
4	133706626	T > C	3	0	rs188750978
5	133706771	T > A	1	0	
6	133706876	T > G	1	0	
7	133706925	A > G	32	0	
8	133706996	T > G	1	0	

**Fig. 2:**
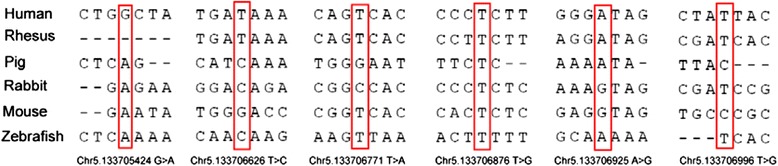
Evolutionary conservation of the promoter sequence of UBE2B affected by variants that are not seen in the controls. Multiple nucleic acid alignments were performed with MegAlign (Demonstration System DNASTAR, Inc.). The identification numbers of the *UBE2B* promoter were as follows: Human (NCBI ID: 7320), Rhesus (NCBI ID: 710678), Pig (NCBI ID: 100513527), Rabbit (NCBI ID: 100009132), mouse (NCBI ID: 22210) and zebrafish (NCBI ID: 437020). The variant alleles are boxed

The other three patient-specific variants (Chr5.133705424 G > A, Chr5133706626 T > C. Chr5. 133706996 T > G) were not in a regulatory sequence analyzed by TRANSFAC software. Alignment of the UBE2B promoter sequence to its orthologs in different species showed that five patient-specific variants (Chr5.133705424 G > A, Chr5133706626 T > C, Chr5.133706771 T > A, Chr5.133706925 A > G and Chr5. 133706996 T > G) were not conserved (Fig. 2). Only one patient-specific variant (Chr5.133706876 T > G) was conserved (Fig. 2).The subjects with variants listed in Table 2 did not carry mutations in the other assayed genes.

### SP1 failed to activate the UBE2B Chr5.133706925 A > G promoter

**Fig. 3:**
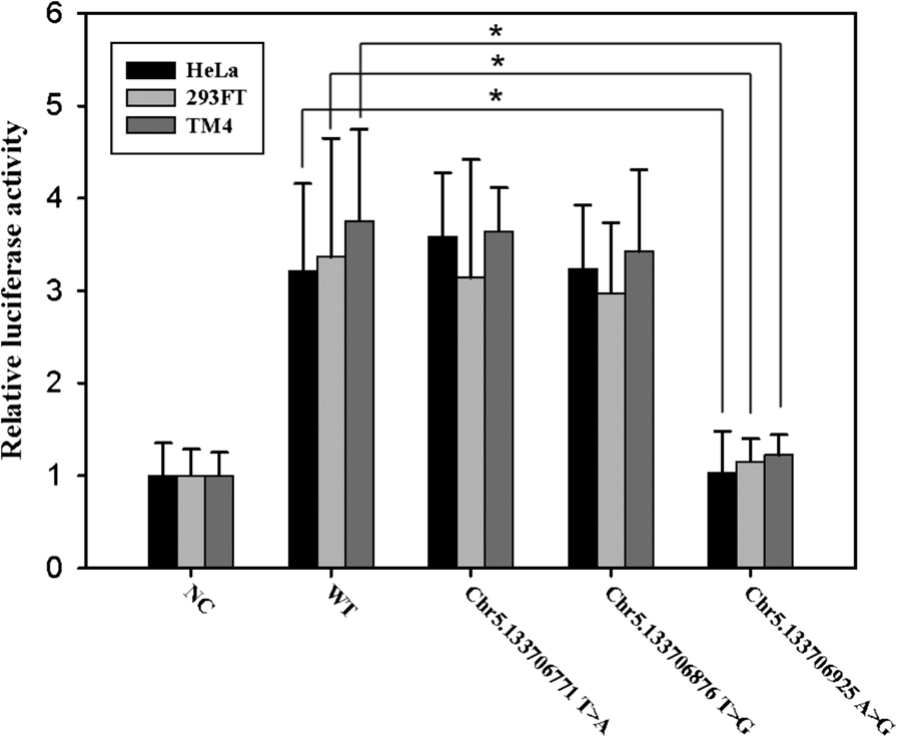
Transactivation of WT and three *UBE2B* mutant promoters by SP1. The pCMV-SP1 and the WT or mutant forms of the *UBE2B* promoter that were cotransfected into HeLa, 293FT and TM4 cells. *UBE2B* promoter activity was analyzed using luciferase assay. Compared with WT and other mutants, SP1 failed to activate the *UBE2B* Chr5.133706925 A > G promoter. The NC: pCMV empty vector was cotransfected with the WT *UBE2B* promoter. Fold induction is shown as the ratio of WT or mutants to the average of NC. (* *P* < 0.01)

To evaluate whether the identified three variants affect the role of SP1 in *UBE2B* promoter activation, luciferase reporter constructs containing the *UBE2B* promoter with these three variants were tested in HeLa, 293FT and TM4 cell lines. The results showed that SP1 significantly increased *UBE2B* promoter WT, Chr5.133706771 T > A, Chr5.133706876 T > G, but not Chr5.133706925 A > G in comparison to the empty vector (Fig. 3).

### Histological analysis

**Fig. 4:**
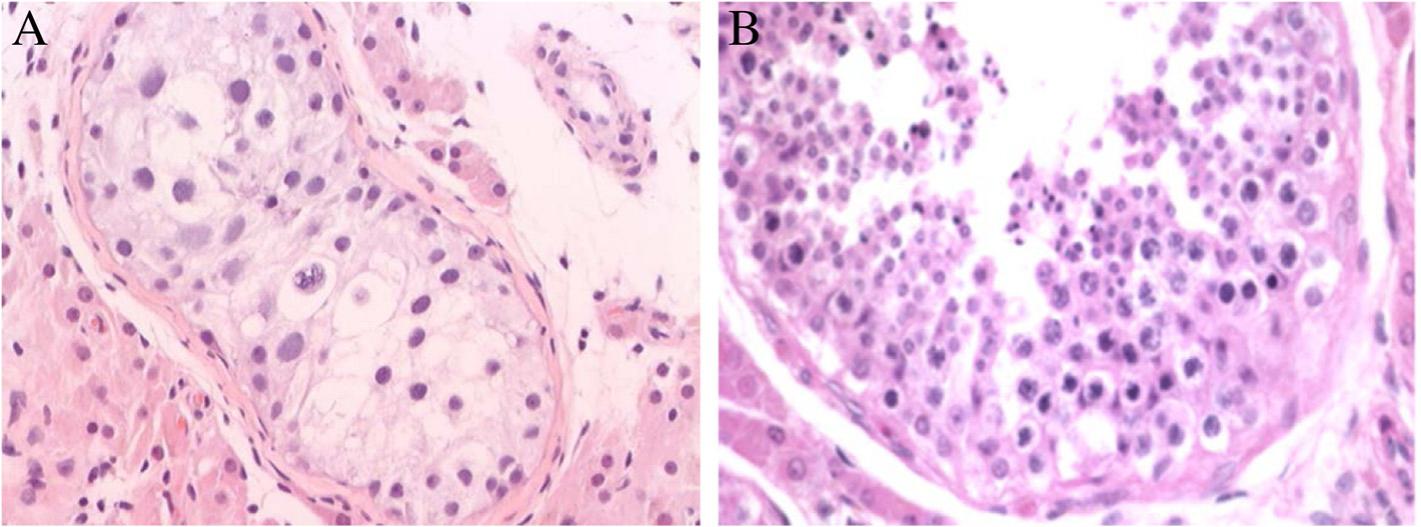
Histopathology of testes from a patient (No. 188) with Chr5.133706925 A > G and normal tissue by hematoxylin and eosin staining. **a**. The patient (No.188). The spermatogenic cells were reduced in contorted seminiferous tubules and there were no mature sperm. **b**. Testis tissue from a patient with obstructive azoospermia as the control. (Magnification: X200)

A patient biopsy (No. 188) with *UBE2B* Chr5.133706925 A > G confirmed the diagnosis of non-obstructive azoospermia. Moreover, we found that spermatogenic cells were significantly reduced in contorted seminiferous tubules (Fig. 4a, b).

## Discussion

Previous studies suggested that azoospermia may result from chromosomal aberrations, microdeletions of the Y chromosome, or single gene variants [20–22]. However, the underlying genes and molecular mechanisms are not well identified.

The UBE2B gene is essential for normal spermatogenesis, and defects in this gene will lead to spermatogenic failure. Matzuk's group found that UBE2B variants exist in oligozoospermic patients, but they did not examine the correlation between UBE2B and IA. In this study, we sequenced the exon and promoter region of *UBE2B* in patients with IA. The Chr5.133706925 A > G promoter variant, located at the SP1-binding sites, was found in 32 of the 776 patients but was absent in 709 fertile men. However, because only the promoter, exons and flanking regions, which are adjacent to the 5' end and 3' end of the gene but not transcribed into RNA, of *UBE2B* gene were sequenced, it remains to be determined whether there are disease-associated variants at intronic splice junctions.

In mammalian spermatogenesis, the histone post-translational modifications such as acetylation, methylation, phosphorylation, sumoylation and ubiquitination have been linked to the regulation of cellular activities such as gene transcription, repair, replication and silencing [23, 24]. UBE2B protein, an ubiquitin-conjugating enzyme, can mediate H2A and H2B ubiquitylation and function during transcription in somatic cells as well as germ cells [25–27]. UBE2B is mainly expressed in round spermatids and Sertoli cells, and it is essential for normal spermatogenesis. *Ube2b*-deficient male mice are infertile as a result of low numbers of predominantly abnormal spermatozoa, abnormal spermatid nuclear condensation, and sperm periaxonemal anomalies [16, 17, 28]. Furthermore, detailed analysis has shown that UBE2B is responsible for proper chromosome crossover in the meiotic recombination of mouse spermatocytes, whereas UBE2B defects lead to arrest of the meiotic prophase and abnormal chromosome synapsis [16, 17, 28]. These results clearly demonstrate the important role of UBE2B in maintaining normal spermatogenesis and the involvement of UBE2B defects in spermatogenic failure. Therefore, it is reasonable to suggest that Chr5.133706925 A > G in the *UBE2B* promoter may affect gene expression by altering the binding sites of some transcription factors, which in turn results in spermatogenic defects.

To provide additional evidence for our conclusion, we performed further experiments to address the biological functional bases underlying the association between *UBE2B* promoter variants and IA risk. First, the sequence region around Chr5.133706925 (A > G) was predicted to be a DNA-binding site of SP1, with the allele A to allele G change leading to the alteration and possibly loss of function for this site. Second, the luciferase assay results showed that substitution of A by G at Chr5.133706925 reduced the regulation of SP1 to the *UBE2B* promoter. In this study, a biopsy of a patient (No. 188) with the *UBE2B* Chr5.133706925 A > G confirmed the diagnosis of non-obstructive azoospermia and further histopathology showed abnormal spermatogenesis. Therefore, these results collectively indicate that Chr5.133706925 A > G in UBE2B promoter confers susceptibility to IA. It is possible that abrogation of the SP1 binding site due to the presence of Chr5.133706925 (A > G) decreased the expression of UBE2B and thus led to spermatogenic failure.

Studies have identified several alternatively spliced transcripts encoding SP1 isoforms that display stage and cell-type-specific expression profiles in differentiating germ cells of the seminiferous epithelium in the testis [29]. Further studies need to be performed to confirm that the GC box of Chr5.133706925 is actually bound by SP1 in spermatids. Additional functional assays are needed to confirm the effects of SP1 on transcription of the UBE2B variant (Chr5.133706925 A > G) and examine the results of the SP1 factor failure to bind in spermatids with this variant.

## Conclusions

In conclusion, with the use of bioinformatics analysis and functional analysis, we identified a variant (Chr5.133706925 A > G) that resides in the *UBE2B* gene promoter and confers a high risk for IA in a Chinese population, possibly through modification of the DNA-binding ability of SP1 and subsequent reduction in UBE2B expression. Our study also demonstrated that systematic analysis of the genetic variants in large cohorts of patients in addition to subsequent functional assays may provide new insights into the cause of IA in humans.

## Abbreviations

IA: Idiopathic azoospermia
UBE2B: Ubiquitin-conjugating enzyme E2B
